# Gli3 controls the onset of cortical neurogenesis by regulating the radial glial cell cycle through *Cdk6* expression

**DOI:** 10.1242/dev.163147

**Published:** 2018-08-20

**Authors:** Kerstin Hasenpusch-Theil, Stephen West, Alexandra Kelman, Zrinko Kozic, Sophie Horrocks, Andrew P. McMahon, David J. Price, John O. Mason, Thomas Theil

**Affiliations:** 1 Centre for Discovery Brain Sciences, Hugh Robson Building, University of Edinburgh, Edinburgh EH8 9XD, UK; 2 Department of Stem Cell Biology and Regenerative Medicine, Eli and Edythe Broad-CIRM Center for Regenerative Medicine and Stem Cell Research, W.M. Keck School of Medicine, University of Southern California, Los Angeles, CA 90033, USA

**Keywords:** *Gli3*, Neurogenesis, Cell cycle, *Cdk6*, Mouse

## Abstract

The cerebral cortex contains an enormous number of neurons, allowing it to perform highly complex neural tasks. Understanding how these neurons develop at the correct time and place and in accurate numbers constitutes a major challenge. Here, we demonstrate a novel role for Gli3, a key regulator of cortical development, in cortical neurogenesis. We show that the onset of neuron formation is delayed in *Gli3* conditional mouse mutants. Gene expression profiling and cell cycle measurements indicate that shortening of the G1 and S phases in radial glial cells precedes this delay. Reduced G1 length correlates with an upregulation of the cyclin-dependent kinase gene *Cdk6*, which is directly regulated by Gli3. Moreover, pharmacological interference with Cdk6 function rescues the delayed neurogenesis in *Gli3* mutant embryos. Overall, our data indicate that Gli3 controls the onset of cortical neurogenesis by determining the levels of *Cdk6* expression, thereby regulating neuronal output and cortical size.

## Abstract (precis)

**Summary:** Examination of conditional mouse mutants reveals a novel role for Gli3 in controlling the cell cycle of radial glial cells and thereby neuron generation and growth of the cerebral cortex.

## INTRODUCTION

The ordered formation of neurons in sufficient numbers and at the correct time and place underlies the functioning of the cerebral cortex and its ability to perform highly complex neural tasks and to confer humans with their unique cognitive capabilities. Central to generating appropriate numbers of neurons are cortical stem and progenitor cells and the control of their proliferation and differentiation rates. Changes in these parameters can have profound effects on cortical size and have been proposed to underlie cortical malformations in human disease as well as the expansion of the human cerebral cortex during evolution (Florio and Huttner, 2014). During murine corticogenesis, there are two major types of stem and progenitor cells: radial glial cells (RGCs) and basal progenitors (BPs), which can be characterized by the expression of the transcription factors Pax6 and Tbr2 (also known as Eomes), respectively (Englund et al., 2005; Götz et al., 1998; Warren et al., 1999). RGCs extend apical and basal processes and present with an apico-basal polarity (Götz and Huttner, 2005). They divide at the apical surface of the ventricular zone to undergo either symmetric proliferative divisions to expand the progenitor pool or asymmetric self-renewing divisions to produce an RGC daughter cell and either a neuron or a BP (Götz and Huttner, 2005). BPs are born from RGCs, have no apical contact and settle in a more basal position to form the subventricular zone. Most BPs divide directly into two neurons, whereas the remainder undergo one round of symmetric proliferative division before differentiating into two neurons (Haubensak et al., 2004; Miyata et al., 2004; Noctor et al., 2004).

The switch from symmetric proliferative divisions to asymmetric divisions in RGCs is crucial to determine neuron numbers and cortical size and is controlled by cell-extrinsic as well as cell-intrinsic mechanisms. Fgf10 and retinoic acid are produced by neuroepithelial cells and by the meninges, respectively, and control the transition between these division modes, thereby regulating cortical neuron formation (Haushalter et al., 2017; Sahara and O'Leary, 2009; Siegenthaler et al., 2009), whereas Notch/Delta signalling between neural progenitors is essential for progenitor self-renewal by regulating the activity of the Hes transcription factors (Kageyama et al., 2008; Pierfelice et al., 2011). Moreover, there is increasing evidence for a pivotal role of the cell cycle as a cell-intrinsic control mechanism to determine the balance between RGC proliferation and differentiation. In particular, the length of the G1 phase correlates with the neurogenic fate of cortical cell divisions. Early during corticogenesis, cortical progenitors have a shorter G1 and undergo proliferative divisions whereas G1 becomes longer for neurogenic divisions at later stages (Miyama et al., 1997). Moreover, G1 lengthening alone is sufficient to induce neurogenesis and shortening G1 inhibits neuron formation (Lange et al., 2009; Pilaz et al., 2009). Although these studies clearly demonstrate the importance of the cell cycle in regulating the switch from proliferative to neurogenic divisions in RGCs, it remains largely unexplored how cell cycle length itself is controlled at the onset of cortical neurogenesis.

The evolutionarily highly conserved zinc-finger transcription factor Gli3 is well positioned to combine roles in early cortical development with the timing of neuron formation. *Gli3* is essential for patterning the telencephalon (Theil et al., 1999; Tole et al., 2000) by repressing Shh signalling and by also acting in a Shh-independent manner (Rash and Grove, 2007). Recent single-cell mRNA-seq experiments identified *GLI3* as an RGC-specific marker in human cortex (Pollen et al., 2015, 2014). *Gli3* has been implicated in murine cortical stem cell development after mid-corticogenesis when it regulates cortical growth (Palma and Ruiz i Altaba, 2004; Wang et al., 2011). Gli3 also helps to establish the adult neurogenic niche by repressing *Il6st* and *Numb* gene expression (Wang et al., 2014). Strikingly, the earliest born cortical neurons are severely reduced and/or completely lost in the *Gli3* mutant forebrain (Magnani et al., 2010, 2013; Theil, 2005), strongly suggesting a role in controlling the transition from symmetric to asymmetric division in RGCs, but the underlying mechanisms remain unexplored. Here, we demonstrate that conditional inactivation of *Gli3* in cortical RGCs leads to a delay in cortical neuron formation that coincides with an increase in cortex size and a reduced proportion of deep layer neurons. Gene expression profiling indicates that altered expression of cell cycle genes precedes this neurogenesis defect. Indeed, the cell cycle length of *Gli3* mutant RGCs is shortened as a result of reduced lengths of the G1 and S phases. Mechanistically, Gli3 binds to the promoter of the *Cdk6* gene, a key regulator of G1 phase length (Choi and Anders, 2014), *in vitro* and *in vivo* and represses *Cdk6* transcription. Interfering with Cdk6 activity rescues the delayed neurogenesis in *Gli3* conditional mutants. Taken together, these findings establish Gli3 as a novel regulator of the RGC cell cycle and show that Gli3 regulates cell cycle length and thereby cortical neurogenesis by controlling *Cdk6* expression.

## RESULTS

### Cortical neurogenesis is delayed in *Gli3* mutant embryos

To address which cortical progenitor cell types express Gli3 protein, we performed Gli3 double immunofluorescence staining with Pax6 and Tbr2 as markers for RGCs and BPs, respectively, on sections of embryonic day (E) 12.5 cortex. This analysis revealed that Gli3 is expressed in Pax6^+^ progenitors. Some Tbr2^+^ cells, mainly located deep within the ventricular zone, also express Gli3 whereas BPs at the upper side of the ventricular zone express little or no Gli3 protein (Fig. S1). These findings indicate that Gli3 is predominantly expressed in RGCs and becomes downregulated in BPs, as has been described for Pax6 (Englund et al., 2005).

**Fig. 1.**
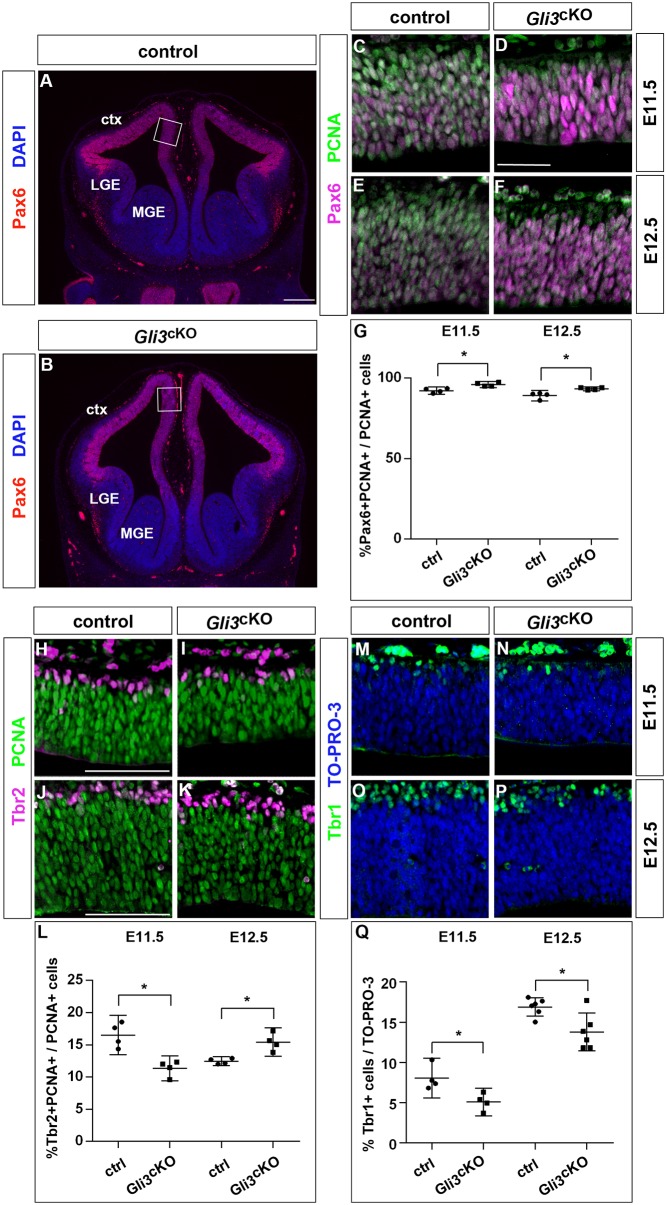
**Altered proportions of RGCs, BPs and neurons in *Gli3* conditional mutants.** (A,B) Coronal sections of E12.5 forebrains stained with DAPI and Pax6 illustrating the overall morphology and the extent of the dorsal telencephalon in *Gli3*^cKO^ mutants. Boxes indicate the region at which cell counts were performed. (C-G) Pax6/PCNA double immunofluorescence staining determines the proportion of RGCs. (G) The fraction of RGCs is increased in E11.5 and E12.5 *Gli3*^cKO^ embryos. (H-L) Tbr2 and PCNA immunostaining on brain sections showed that the proportion of BPs is decreased at E11.5 but increased at E12.5. (M-Q) Reduced proportions of neurons in the rostromedial telencephalon of *Gli3*^cKO^ embryos as revealed by staining for Tbr1 and TO-PRO-3. All statistical data are presented as mean±95% confidence intervals (CI); *n*=4 (C-F,H-K,M,O) and *n*=6 (N,P); *P<0.05; Mann–Whitney test. ctx, cortex; LGE, lateral ganglionic eminence; MGE, medial ganglionic eminence. Scale bars: in A, 250 µm for A,B; in D, 25 µm for C-F; in H, 50 µm for H-K,M-P.

Given its expression in RGCs, *Gli3* could regulate their proliferation or their differentiation into BPs and cortical projection neurons. To investigate such roles, we made use of *Emx1Cre*;*Gli3*^fl/fl^ (*Gli3*^cKO^) conditional mutants. In these embryos, *Gli3* is inactivated in the cortex in a gradient from medial to lateral with inactivation being completed medially by E11.5 with the onset of neurogenesis. In contrast, Gli3 protein expression in the lateral neocortex is only lost by E12.5 when neurogenesis is already underway (Fig. S1). Moreover, E12.5 *Gli3*^cKO^ embryos can easily be distinguished from control embryos by a prominent bulging of the rostral telencephalon suggesting a severe growth defect (Fig. S2). Coronal sections through the telencephalon revealed an elongation of the rostral midline and a thinner cortex with lower numbers of cells per unit of surface but no obvious defect in cortical patterning as described previously (Amaniti et al., 2013) (Fig. 1A,B; Fig. S3). Given this growth defect, we focussed our analysis of RGC development on the rostromedial telencephalon of *Gli3*^cKO^ embryos. We first determined the proportions of RGCs, BPs and cortical neurons in this region in E11.5 and E12.5 *Gli3*^cKO^ embryos, i.e. at the beginning of cortical neurogenesis. Double immunofluorescence staining for PCNA, a marker of proliferating cells (Hall et al., 1990), and the radial glial marker Pax6 revealed an increase in the proportion of RGCs in E11.5 and E12.5 *Gli3*^cKO^ embryos (Fig. 1C-G). This rise coincided with a decreased proportion of proliferating BPs and neurons at E11.5 as evidenced by Tbr2/PCNA and Tbr1/TO-PRO-3 double staining, respectively (Fig. 1H,I,L-N,Q). In contrast, the BP pool was increased by E12.5, although the proportion of neurons remained decreased (Fig. 1J-L,O-Q). Taken together, these findings indicate that the formation of BPs and cortical neurons is delayed in *Gli3*^cKO^ embryos, suggesting that Gli3 controls the timing of RGC differentiation into BPs and neurons.

### Reduced cell cycle exit and decreased formation of deep layer neurons in *Gli3*^cKO^ mutants

**Fig. 2.**
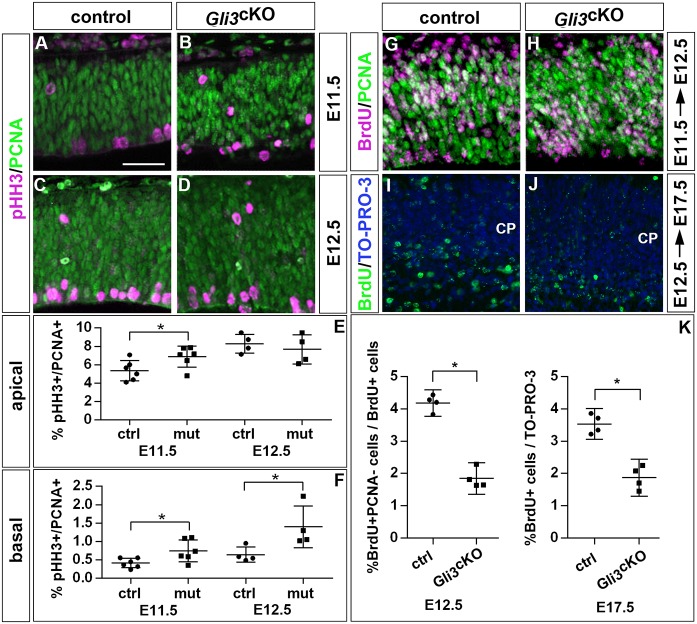
**Increased proliferation and reduced cell cycle exit in *Gli3*^cKO^ mutants.** (A-D) Sections of E11.5 and E12.5 control and *Gli3*^cKO^ embryos stained with pHH3/PCNA. (E,F) Quantification of the data presented in A-D showing increased proportions of RGCs (E11.5) and BPs (E11.5 and E12.5) undergoing mitosis. (G,H) BrdU/PCNA immunohistochemistry on sections of E12.5 control and *Gli3*^cKO^ embryos that were treated with BrdU 24 h earlier. (I,J) BrdU immunofluorescence staining on sections of E17.5 control (I) and *Gli3*^cKO^ embryos (J) to reveal neurons born at E12.5. Only the cortical plate (CP) is shown. (K) Quantification of the data presented in G-J showing decreased cell cycle exit and neuron formation in *Gli3*^cKO^ embryos. All statistical data are presented as mean±95% CI; *n*=6 (A,B) and *n*=4 (C,D,G-J); *P<0.05; Mann–Whitney test. Scale bar: in A, 25 µm for A-D,G-J.

To determine whether reduced neuron numbers in *Gli3* mutants were due to increased neural progenitor proliferation, we performed double immunofluorescence experiments for PCNA and phosphohistone H3 (pHH3), which labels mitotic RGCs at the ventricular surface and dividing BPs in abventricular positions. This analysis confirmed increased proportions of RGCs and BPs undergoing mitosis in E11.5 *Gli3*^cKO^ embryos (Fig. 2A,B,E,F). This effect is also maintained in E12.5 mutant BPs but not in RGCs (Fig. 2C-F). We also investigated the possibility of altered cell cycle exit using bromodeoxyuridine (BrdU) pulse-chase experiments. BrdU was given to pregnant mice 24 h before dissecting the embryos and PCNA staining was used to reveal all proliferating cells. This analysis showed that the fraction of cells leaving the cell cycle and differentiating into neurons (BrdU^+^/PCNA^−^) was significantly reduced in E12.5 *Gli3*^cKO^ embryos, consistent with the reduced proportions of neurons in these areas (Fig. 2G,H,K). This analysis suggests that alterations in proliferation and cell cycle exit underlie the changes in neuronal proportions in *Gli3* mutants.

**Fig. 3.**
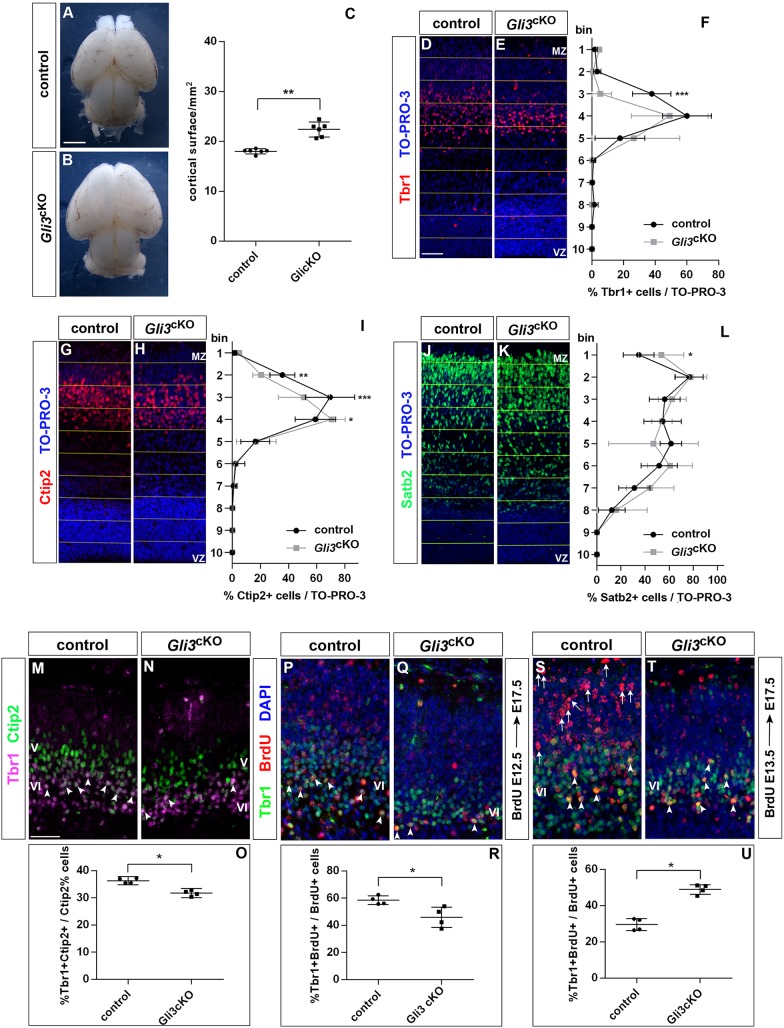
***Gli3* conditional inactivation affects cortical size and architecture.** (A,B) Dorsal views of E18.5 control (A) and *Gli3*^cKO^ (B) brains. *Gli3*^cKO^ embryos only form a small olfactory bulb-like structure in the rostral telencephalon, which is not visible in this dorsal view (Amaniti et al., 2015). (C) Graph comparing the surface area of control and *Gli3*^cKO^ cerebral cortices. (D-L) Immunohistochemistry on E17.5 control and *Gli3*^cKO^ brains using the indicated antibodies. (D-F) Tbr1^+^ neurons occupy a reduced area in the deep cortical plate of the *Gli3*^cKO^ cortex. (G-I) Ctip2^+^ neurons occupy deeper positions in the cortical plate of *Gli3*^cKO^ embryos. (J-L) Distribution of Satb2^+^ upper layer neurons, which have not completed their migration at this stage. (M-O) Tbr1 and Ctip2 double immunofluorescence on coronal sections of E17.5 brains showed that the relative position of Tbr1^+^ layer VI and Ctip2^+^ layer V neurons is maintained in *Gli3*^cKO^ embryos. Moreover, the proportion of Tbr1^+^Ctip2^+^ double positive neurons (arrowheads) is reduced in the mutant (O). (P-U) The formation of Tbr1^+^ neurons is delayed in *Gli3*^cKO^ embryos. (P-R) The proportion of Tbr1^+^ neurons born at E12.5 (arrowheads) is reduced in *Gli3*^cKO^ embryos. (S-U) BrdU birthdating at E13.5 showed an increase in BrdU^+^Tbr1^+^ neurons (arrowheads) in *Gli3*^cKO^ embryos. Note the large number of BrdU^+^Tbr1^−^ neurons (arrows) superficial to the Tbr1 domain in control embryos. All statistical data are presented as mean±95% CI; *n*=6 (A-C) and *n*=4 (D-U); *P<0.05; **P<0.01; ***P<0.005; Mann–Whitney test (C,O,R,U) and two-way ANOVA followed by Sidak's multiple comparisons test (F,I,L). MZ, marginal zone; VZ, ventricular zone. Scale bars: in A, 2 mm for A,B; in D,M, 50 µm for D,E,G,H,J,K,M,N,P,Q,S,T.

Next, we investigated the consequences of this delayed cortical neurogenesis on cortical size and layer formation. We predicted that a delay in early neuron generation would result in an enlarged cortex and in reduced proportions of deep layer neurons. We first confirmed a reduction in the formation of early-born neurons in the *Gli3*^cKO^ cortex using a BrdU pulse-labelling experiment in which BrdU was injected into pregnant dams at the E12.5 stage and embryos were recovered at E17.5 (Fig. 2I-K). Next, we measured cortical surface area, which was significantly increased in *Gli3*^cKO^ brains (Fig. 3A-C). Measurements on coronal sections also revealed an enlarged ventricular surface and a thicker cortex in *Gli3*^cKO^ mutants (Fig. S4). We also used immunofluorescence labelling for Tbr1 and Ctip2 (also known as Bcl11b) to investigate the formation of layer VI and layer V neurons, respectively, whereas Satb2 served as a layer II-IV neuronal marker. To this end, the neocortex was subdivided into ten bins (bin ten being the deepest) and the proportions, distribution and number of each neuronal subtype were determined. This analysis revealed a reduced number and proportion of early-born Tbr1^+^ neurons (Fig. S5) which settled in a narrower band (Fig. 3D-F). This reduced size of the Tbr1^+^ layer coincided with a shift of Ctip2^+^ neurons to a deeper location (Fig. 3G-I). The proportion of Tbr1^+^Ctip2^+^ neurons mainly located in layer VI was also decreased (Fig. 3M-O) and birthdating analyses showed a delayed generation of Tbr1^+^ neurons in *Gli3*^cKO^ mutants (Fig. 3P-U). In contrast, we observed an increase in the proportion and numbers of Satb2^+^ neurons (Fig. 3J-L) (Fig. S5). Taken together, these experiments led us to conclude that a reduced cell cycle exit at early neurogenesis led to an increased cortical size and to the reduced and delayed formation of Tbr1^+^ deep layer neurons in *Gli3*^cKO^ mutants.

### Gli3 regulates the RGC cell cycle

To investigate the molecular mechanisms by which Gli3 regulates RGC differentiation, we compared gene expression profiles in control and *Gli3*^cKO^ embryos by mRNA sequencing (mRNA-seq). To this end, we dissected the rostromedial telencephalon from embryos at E11.5 when the delay in BP and neuron formation started and at E12.5 when BPs and neurons were formed. We found that 2515 and 458 genes were differentially regulated in *Gli3*^cKO^ embryos compared with control embryos at E11.5 and E12.5, respectively (*P*<0.05) (Table S3). Gene ontology (GO) analysis using Genomatix Software showed that differentially regulated genes are mostly involved in cell cycle regulation at E11.5 (GO terms: ‘mitotic cell cycle’, ‘cell cycle phases’ and ‘G1/S transition’) and in neuronal differentiation at E12.5 (GO terms: ‘generation of neurons’ and ‘neuronal differentiation’) (Fig. S6). Other regulated genes were involved in ‘DNA replication’ and ‘nervous system development’ at E11.5 and in ‘cell differentiation’ and ‘forebrain development’ at E12.5, consistent with known roles of Gli3 in cortical development.

**Fig. 4.**
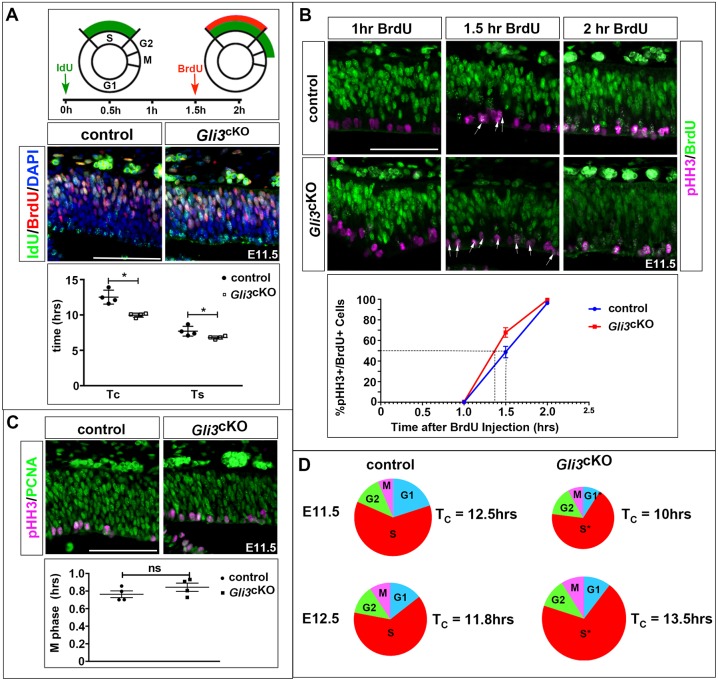
**Cell cycle length of cortical progenitors is affected in *Gli3*^cKO^ embryos.** (A) BrdU/IdU double labelling experiments to determine total cell cycle length (T_C_) and S-phase duration (T_S_), which were calculated from counts of IdU^+^BrdU^−^ and IdU^+^BrdU^+^ cells as described (Martynoga et al., 2005). E11.5 *Gli3*^cKO^ embryos show a shortening of T_C_ and T_S_. The schematic illustrates the timing of IdU and BrdU injections and the progression of labelled cells in the cell cycle. (B) BrdU labelling experiments to investigate the duration of G2 (T_G2_). Double labelling for BrdU and pHH3 determined the proportion of BrdU^+^/pHH3^+^ cells (arrows). T_G2_ corresponds to the time when 50% of pHH3^+^ cells are BrdU^+^ (indicated by the dotted lines) and is not significantly altered between E11.5 control and *Gli3*^cKO^ embryos. (C) Determining M-phase length (T_M_) using immunostaining for pHH3 and PCNA, which label mitotic and proliferating cells, respectively. The fraction of mitotic cells multiplied by total cell cycle length provides T_M_ duration, which is not significantly altered in E11.5 *Gli3*^cKO^ embryos. (D) Pie charts summarizing the length of total and individual cell cycle phases in E11.5 and E12.5 control and *Gli3*^cKO^ embryos. Shorter G1 and S phases contribute to a shortening of the overall cell cycle at E11.5 whereas T_C_ is increased at E12.5 due to longer S and M phases. All statistical data are presented as mean±95% CI; n=4; *P<0.05; Mann–Whitney test. ns, not significant. Scale bars: 50 µm.

The above findings indicate that alterations in the cell cycle of RGCs precede and may underlie defects in neurogenesis in *Gli3*^cKO^ cortical progenitors. To test this idea, we first determined total cell cycle length and that of individual cell cycle phases in control and *Gli3*^cKO^ embryos. We used iododeoxyuridine (IdU) and BrdU double labelling to calculate total cell cycle and S-phase times (Fig. 4A) (Martynoga et al., 2005; Nowakowski et al., 1989). In addition, we combined BrdU labelling with immunostaining for pHH3, an indicator of late G2 and M phase, to determine G2 length (Fig. 4B). To this end, embryos were harvested at different time points after BrdU administration to identify when BrdU label appears in mitotic figures. M-phase length was investigated by analysing the proportion of progenitors that are in M phase by pHH3 immunofluorescence compared with the total number of progenitors as revealed by PCNA staining (Fig. 4C). Finally, determining total cell cycle length and length of S, G2 and M phase allowed us to calculate the length of the G1 phase. These analyses revealed a shortening of the overall cell cycle in E11.5 *Gli3*^cKO^ cortical progenitors coinciding with significantly shorter G1 and S phases (Fig. 4D, Table 1 and Table S4). In contrast, the cell cycle was elongated due to a lengthening of S phase in E12.5 *Gli3*^cKO^ embryos (Fig. 4D, Table 1 and Table S4). Taken together, these analyses revealed altered cell cycle times in *Gli3*^cKO^ mutants indicating that Gli3 has an important role in controlling the length of the G1 and S phases.

**Table 1.**
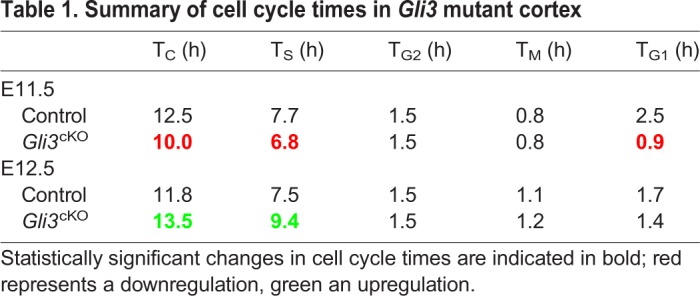
**Summary of cell cycle times in *Gli3* mutant cortex**

### Proportion of proliferating versus differentiating progenitors and changes in S-phase length in *Gli3* mutants

Next, we investigated the causes for the changes in S-phase length in *Gli3* mutants. As transcription during S phase is limited, we addressed whether *Gli3* controls duration of S phase by an indirect mechanism. Recently, it was shown that differentiating RGCs and BPs have a shorter S phase than proliferative progenitors (Arai et al., 2011) raising the possibility that the shortening of S phase in *Gli3* mutants is caused by a higher proportion of progenitors that are differentiating. These cells are characterized by *Btg2* (*Tis21*) expression (Iacopetti et al., 1999). Therefore, we generated *Gli3* mutants carrying a Tis21-GFP transgene (Haubensak et al., 2004). Double immunofluorescence for GFP and Pax6 revealed no statistically significant change in the proportion of differentiating RGCs in E11.5 *Gli3*^cKO^ embryos but a decrease in E12.5 RGCs (Fig. S7). In contrast, the proportion of differentiating BPs was slightly increased at E11.5 (Fig. S7). However, as BPs only form a small proportion of the overall cortical progenitor population in the dorsomedial telencephalon, it is unlikely that this change contributes to the changes in S-phase length in *Gli3*^cKO^ mutants.

### Gli3 directly represses *Cdk6* expression

As a next step, we analysed the mechanisms by which Gli3 regulates G1 duration. Among the cell cycle-regulated genes, cyclin-dependent kinase 6 (*Cdk6*) was the most highly upregulated gene in *Gli3*^cKO^ embryos compared with controls (1.9-fold) (Table S3). Cdk6 has a key role in controlling the transition between the G1 and S phases of the cell cycle and thereby determines the length of the G1 phase (Choi and Anders, 2014). The shorter G1 phase and its upregulation in *Gli3* mutant cortical progenitors made *Cdk6* an interesting candidate acting downstream of *Gli3* to control the length of the G1 phase.

**Fig. 5.**
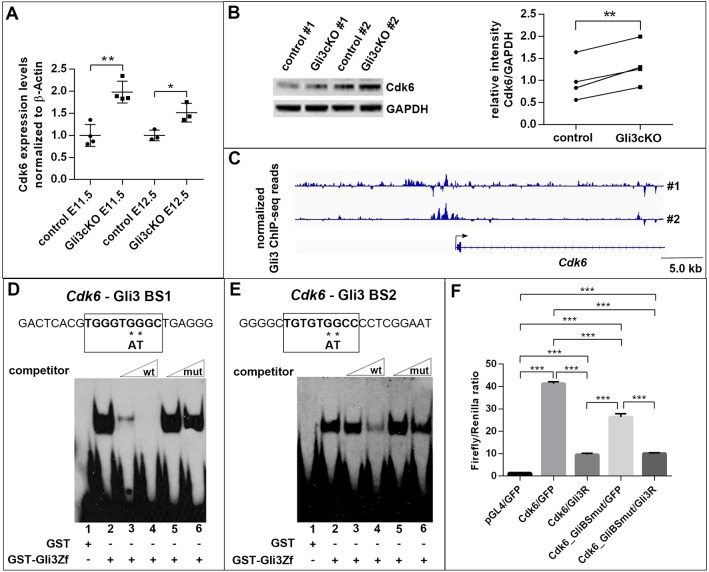
**Gli3 represses *Cdk6* expression.** (A) Representative example of qRT-PCR analyses showing increased *Gli3* mRNA expression in the rostromedial telencephalon of E11.5 and E12.5 *Gli3*^cKO^ embryos. (B) Western blot analysis showing upregulation of Cdk6 expression in the dorsal telencephalon of E12.5 of *Gli3*^cKO^ embryos. Data expressed as Cdk6 expression relative to GAPDH, which served as a loading control. A paired *t*-test was used to evaluate relative Cdk6 expression levels in four control/*Gli3*^cKO^ embryo pairs derived from four different litters. (C) Gli3 chromatin immunoprecipitation followed by sequencing revealed several peaks that are associated with the *Cdk6* promoter region. Gli3 ChIP-seq reads were normalized to input. (D,E) Electromobility shift assays showing *in vitro* binding of recombinant Gli3 protein to binding sites 1 (D) and 2 (E) in the *Cdk6* promoter (lane 2). Complex formation is competed by increasing amounts of wild-type oligonucleotide (lanes 3 and 4) but not by oligonucleotides containing the indicated point mutations in the Gli3-binding sites (lanes 5 and 6). (F) Gli3 represses *Cdk6* promoter activity in luciferase assays. Firefly luciferase activity was measured relative to *Renilla* luciferase control in HEK293 cells transfected with the indicated constructs. pGL4 is the promoterless vector used to make the *Cdk6* promoter construct (Cdk6), which was either co-transfected with a GFP expression vector (GFP) or with a Gli3 repressor (Gli3R) construct. Data are representative of one of three independent experiments each using three technical replicates. All statistical data are presented as mean±95% CI; *n*=4 (A E11.5 embryos,B); *n*=3 (A E12.5 embryos,F); *P<0.05; **P<0.01; ***P<0.001; unpaired t-test (A); paired t-test (B); two-way ANOVA followed by Tukey multiple comparisons tests (F).

We first validated the *Cdk6* upregulation by quantitative RT-PCR, which confirmed a 1.95- and 1.51-fold upregulation of *Cdk6* transcription in the rostromedial telencephalon of E11.5 and E12.5 *Gli3*^cKO^ embryos, respectively, (Fig. 5A) consistent with our mRNA-seq data. Western blotting revealed a 1.35-fold upregulation of the Cdk6 protein in the dorsal telencephalon of E12.5 *Gli3*^cKO^ embryos (Fig. 5B).

Next, we investigated the mechanism by which Gli3 regulates *Cdk6* expression. The upregulation of *Cdk6* in *Gli3*^cKO^ mutants raises the possibility that Gli3 binds to the *Cdk6* promoter and directly represses its transcription. Chromatin immunoprecipitation followed by deep sequencing identified several sites of Gli3 occupancy in the *Cdk6* promoter region (Fig. 5C). Inspecting the promoter sequence revealed two potential, evolutionarily conserved Gli3-binding sites. Using electromobility shift assays (EMSAs), we tested whether these sites can specifically bind a GST-Gli3 protein containing the Gli3 DNA-binding domain. Incubation of GST-Gli3 with oligonucleotides containing the Gli-binding motif from the *Cdk6* promoter resulted in the formation of a slower migrating complex (Fig. 5D,E). Competition assays in the presence of various amounts of surplus unlabelled wild-type oligonucleotide (competitor) resulted in progressively diminished binding of GST-Gli3 fusion protein with increasing amounts of competitor (Fig. 5D,E). However, complex formation was not competed by unlabelled *Cdk6* competitor oligonucleotide containing a GG to AT exchange (Fig. 5D,E), which abolishes Gli binding (Hepker et al., 1999), suggesting that Gli3 can specifically bind to sequences within the *Cdk6* promoter.

We further explored the functionality of the Gli3-binding sites using luciferase reporter assays in HEK293 cells. We cloned a 3.35 kb fragment immediately upstream of the *Cdk6* transcriptional start site into the promoterless luciferase reporter plasmid pGL4.10. Transfecting this construct into HEK293 cells resulted in robust induction of luciferase activity (Fig. 5F). However, co-transfection of the *Cdk6* promoter/luciferase reporter construct with a Gli3 repressor expression plasmid (Persson et al., 2002) strongly reduced reporter gene expression. In contrast, mutations in the Gli3-binding sites led to strong promoter activity (Fig. 5F) but levels were lower than for the wild-type construct and the promoter could still be repressed by co-transfection of a Gli3 repressor construct, potentially due to non-consensus Gli-binding sites (Vokes et al., 2008). Taken together, these analyses demonstrate that Gli3 can specifically bind to sequences in the *Cdk6* promoter region and repress *Cdk6* promoter activity.

### Interfering with Cdk6 function restores the onset of neurogenesis in *Gli3*^cKO^ mutants

**Fig. 6.**
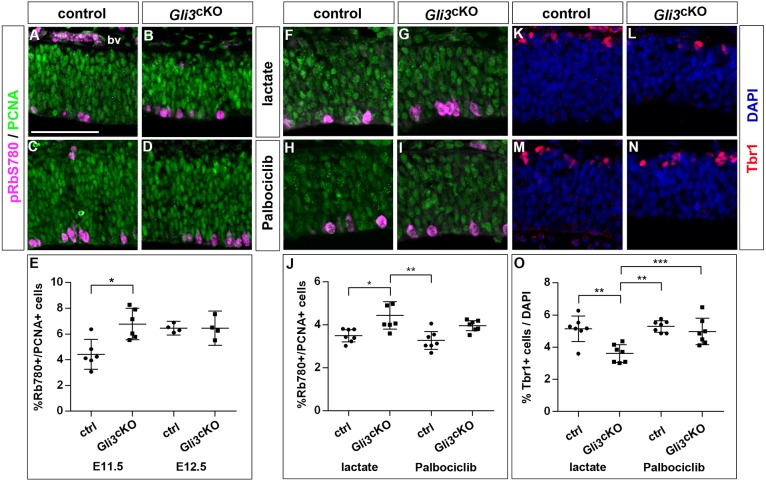
**Interfering with Cdk6 kinase activity rescues the early delay in neurogenesis in *Gli3*^cKO^ embryos.** (A-D) Immunohistochemistry showing expression of pRb-S780 and PCNA in E11.5 (A,B) and E12.5 (C,D) control and *Gli3*^cKO^ embryos. (E) Quantification of the proportion of pRb-S780^+^/PCNA^+^ cells, which is increased in *Gli3*^cKO^ embryos at E11.5. (F-I) Immunostaining for pRb-S780 and PCNA in E11.5 control and *Gli3*^cKO^ embryos 24 h after treatment with lactate as vehicle or with the Cdk6 inhibitor palbociclib. (J) Quantification of the pRb-S780^+^/PCNA^+^ cell proportion after treatment with lactate or palbociclib. (K-N) Immunohistochemistry for Tbr1 revealing the proportions of neurons in E11.5 control and *Gli3*^cKO^ embryos after lactate or palbociclib administration. (O) Quantification of the immunostaining in K-N. All statistical data are presented as mean±95% CI; *n*=4 (B,D); *n*=6 (A,C); *n*=7 (F-I,K-N); *P<0.05*; **P<0.01; ***P<0.005; Mann–Whitney test (E); ANOVA with Tukey multiple comparisons test (J,O). bv, blood vessels. Scale bar: in A, 50 µm for A-D,F-I,K-N.

Next, we investigated a potential role of *Cdk6* as a Gli3 downstream target to control the timing of cortical neurogenesis. Activated Cdk6 phosphorylates a number of proteins, including the retinoblastoma (Rb) proteins, which upon their hyperphosphorylation release the E2F transcription factors from inactive Rb/E2F complexes. E2F factors activate the G1-S transcriptional programme necessary for progression into S phase (Choi and Anders, 2014). Many of the genes characteristic of this programme, including *Cdc6*, *Mcm2-7*, *Cdt1*, the E2Fs *E2f1* and *E2f2* and cyclin E1 and cyclin E2 are upregulated in E11.5 *Gli3*^cKO^ embryos. We also examined the phosphorylation status and the distribution of the Rb protein in *Gli3* mutant cortical progenitor cells. Cyclin/Cdk complexes are known to phosphorylate Rb at specific residues including Ser-780 (Ely et al., 2005; Kitagawa et al., 1996; Zarkowska and Mittnacht, 1997). In the rostromedial telencephalon of E11.5 and E12.5 embryos, most pRb-S780-positive cells were located at the ventricular surface where RGCs undergo mitosis before they enter G1 phase. In E11.5 *Gli3*^cKO^ embryos, the proportion of pRb-S780-positive cells with respect to the total number of proliferative cells was increased but there was no significant change at E12.5, which is consistent with the shortening and elongation of the cell cycle at E11.5 and E12.5, respectively (Fig. 6A-E). Overall, these data indicate that the upregulation of *Cdk6* in *Gli3* conditional mutants coincides with an increased phosphorylation of Rb at Ser780 in cortical progenitor cells and with an upregulation of the G1-S transcriptional programme, suggesting that increased levels of *Cdk6* transcription prematurely drive progenitor cells into proliferation.

Finally, we directly tested whether the upregulation of *Cdk6* underlies the delayed cortical neurogenesis in *Gli3*^cKO^ embryos. To this end, we made use of palbociclib, which selectively inhibits Cdk4/6 with high specificity (Fry et al., 2004) and treated E10.5 pregnant mice either with lactate buffer as vehicle or with a single dose of 75 mg palbociclib/kg body weight before analysing embryos 24 h later. This dose has previously been shown to reduce Cdk4/6 activity in embryos but not to completely block Cdk4/6 function (Fry et al., 2004). In control experiments, we first determined the effects of these treatments on the phosphorylation of Rb as a direct target of the Cdk4/6 kinases. Immunohistochemistry confirmed that the proportion of pRb-S780-positive cells relative to the total number of PCNA^+^ proliferative cells was increased in *Gli3*^cKO^ embryos treated with vehicle but did not differ between control and *Gli3*^cKO^ embryos after palbociclib treatment (Fig. 6F-J), indicating that this regimen resulted in the reduction of Cdk4/6 activity to control levels. Next, we analysed the formation of cortical neurons. Under control conditions, the proportion of neurons was reduced in *Gli3*^cKO^ embryos, consistent with our previous findings (Fig. 6K,L,O). In contrast, treating with palbociclib restored the proportion of cortical neurons (Fig. 6M-O). We also tested whether Cdk6 inhibition at a later stage could also affect cortical neurogenesis in *Gli3*^cKO^ embryos. To this end, we repeated the palbociclib regimen with E11.5 pregnant mice, which were also injected with a single dose of BrdU, and determined the proportion of newly born Tbr1^+^BrdU^+^ neurons 24 h later. This analysis revealed a slight increase in the proportion of newly formed neurons in *Gli3*^cKO^ embryos under control conditions; however, this was not further increased after palbociclib treatment (Fig. S8). Taken together, these findings strongly support the hypothesis that Cdk6 acts as a downstream target of Gli3 to control the onset of neuron formation in the cortex.

## DISCUSSION

Patterning and growth of the developing cerebral cortex have to be tightly coordinated to allow for the formation of appropriate types and numbers of neurons in time and space. Here, we show that the transcription factor Gli3, which has previously been implicated as a key regulator of cortical patterning, has an additional role in controlling the onset of cortical neurogenesis. Cortex-specific inactivation of *Gli3* resulted in the delayed formation of neurons. This defect is caused by a shortening of the cell cycle and in particular the lengths of G1 and S phases. Moreover, Gli3 negatively regulates *Cdk6* expression levels and pharmacological interference with Cdk6 function rescues the early delay in neurogenesis caused by cortical *Gli3* inactivation, indicating that Gli3 controls early cortical neurogenesis by regulating *Cdk6* expression levels.

Controlling the onset of neurogenesis represents a key requirement for forming a cortex of the appropriate size, but how this control is achieved remains largely unknown. Here, we show that *Gli3* inactivation in cortical progenitors led to a delay in the formation of the earliest cortical neurons, indicating a novel role for Gli3 in controlling the onset of neurogenesis. Interestingly, a previous study using *NestinCre*;*Gli3* conditional mutants identified a bias towards the formation of deep layer neurons at the expense of upper layer neurons (Wang et al., 2011). These different findings might be explained by differences in the timing of *Gli3* inactivation, indicating temporal-specific roles of *Gli3* at the onset of neurogenesis and at later neurogenic stages. A time-dependent function in cortical neuron formation is also supported by our palbociclib experiments.

Gene expression profiling led us to hypothesize that changes in cell cycle regulation underlie the delayed neurogenesis in the *Gli3* mutants. This idea is consistent with previous findings implicating the cell cycle as a pivotal regulator of cortical neurogenesis. In particular, the length of the G1 phase plays a major role. It increases during neurogenesis and artificial lengthening of G1 leads to premature neurogenesis (Calegari and Huttner, 2003; Lange et al., 2009; Pilaz et al., 2009). G1 length is mainly regulated by Cdk4/6/cyclin D complexes, which form a central node in a complex signalling network that promotes the G1-S transition (Choi and Anders, 2014). Activation of the Cdk4/6 kinase, which is controlled by a multitude of signalling cues converging on the Cdk4/6 node, leads to Rb hyperphosphorylation and subsequent release of the E2F transcription factors. These factors in turn activate the G1/S transcriptional programme, which includes the expression of genes encoding proteins that are involved in the formation of the pre-recombination complex, DNA synthesis and DNA repair (Choi and Anders, 2014).

We present multiple lines of evidence that Gli3 controls the G1 length at the onset of neurogenesis by directly regulating *Cdk6* expression levels. Cortex-specific inactivation of *Gli3* led to a shortening of G1 at the start of neurogenesis, to an upregulation of *Cdk6* and to a concomitant activation of genes of the G1/S transcriptional programme. We also show that Gli3 binds to the *Cdk6* promoter *in vitro* and *in vivo* as in the developing limb (Lopez-Rios et al., 2012; Vokes et al., 2008), and that its activity is repressed by Gli3. Finally, pharmacological interference with Cdk6 kinase activity using the highly specific inhibitor palbociclib (Fry et al., 2004) specifically rescued the delayed start of neurogenesis in *Gli3*^cKO^ embryos but had no effect at a later neurogenic stage. Taken together, these findings strongly implicate Gli3 as a direct regulator of *Cdk6* and hence G1 length in RGCs. Interestingly, Pax6 also controls *Cdk6* transcription (Mi et al., 2013) and essential Pax6-binding sites are in close proximity to the Gli3-binding sites (K.H.-T. and T.T., unpublished) raising the possibility that both transcription factors might interact to control *Cdk6* expression levels in RGCs. Thus, in addition to post-translational control of Cdk6 kinase activity, controlling *Cdk6* mRNA levels constitutes an important regulatory mechanism to fine-tune the balance between proliferation and differentiation in cortical RGCs.

Though delayed, the switch from proliferative to neurogenic divisions eventually occurs in *Gli3* mutant RGCs. Despite *Cdk6* still being upregulated in E12.5 *Gli3*^cKO^ embryos, the cell cycle is significantly longer with no change in G1 phase length compared with control embryos. Also, Rb-S780 phosphorylation is no longer increased. To promote G1 progression, Rb phosphorylation must surpass a threshold (Blomen and Boonstra, 2007). This phosphorylation, however, depends on the sequential activity of Cdk4/6/cyclin D and Cdk2/cyclin E complexes, which are negatively regulated by the Cdk inhibitors Cdkn1a, b and c (Lim and Kaldis, 2013). According to our gene expression profiling, these inhibitors are only downregulated in E11.5 but not in E12.5 *Gli3*^cKO^ embryos. Therefore, the activity levels of the Cdk complexes might not be sufficient to surpass the Rb phosphorylation threshold at E12.5, which might also explain the time-limited effect of the palbociclib treatment. The resulting prolonged cell cycle, a characteristic feature of differentiative divisions (Calegari et al., 2005; Caviness et al., 1995; Takahashi et al., 1995), could indicate a compensatory switch from self-renewing to neurogenic divisions corresponding with the increased neuron formation in the *Gli3* mutants at the later stage.

Although evaluating the role of the G1 phase has gained much attention, recent work suggested the importance of S phase in cortical development. Cortical stem and progenitor cells shorten S phase on commitment to neuron production with the length of other cell cycle phases remaining unaltered (Arai et al., 2011). S-phase length is also the most distinguishing feature between progenitor cell populations in ferret cortex (Turrero García et al., 2016). A stronger requirement for DNA repair and replication fidelity in proliferative versus differentiating progenitors is thought to underlie a longer S phase, suggesting that S phase is a key factor in maintaining the proliferative capacity of neural progenitor cells (Arai et al., 2011; Soufi and Dalton, 2016). Despite this pivotal role, it remains largely unknown whether and how S-phase duration is controlled by cortical transcription factors. Interestingly, *Gli3* cortical inactivation led to a shorter S phase in cortical progenitors. This change is not due to a shift in the proportion from proliferating to differentiating progenitors, which have a shorter S phase (Arai et al., 2011), or due to a change in the proportions of apical progenitors and BPs. Rather, S-phase shortening is likely to reflect an intrinsic change of *Gli3* mutant RGCs as these cells make up the vast majority of progenitors in the E11.5 rostromedial telencephalon. The consequences of this S-phase shortening are currently unclear but, based on the above model, one would predict an increase in neurogenesis, which we do observe 1 day later. Thus, the shorter S phase might be an early indicator of a switch towards asymmetric neurogenic divisions.

There are several mutually non-exclusive possibilities for how Gli3 could regulate S-phase duration. First, the upregulation of the G1/S programme may allow for faster progression through S phase. Second, Gli3 might directly control the expression of genes determining S-phase length. Indeed, an upregulation of cyclin A2, which regulates S-phase progression (Yam et al., 2002), in combination with a reduced expression of the cyclin inhibitors *Cdkn1a*, *b* and *c* could induce RGCs to progress faster through S phase. Finally, S phase involves the control of DNA quality and repair and a large number of DNA repair genes are upregulated (differentially expressed) in E11.5 *Gli3*^cKO^ mutants. Interestingly, there is a large overlap in this set of Gli3-regulated genes with E2F target genes (Ren et al., 2002). As E2F genes are upregulated in *Gli3*^cKO^ embryos, this finding raises the interesting possibility that Gli3 regulates S-phase length in RGCs by controlling the expression levels of the E2F transcription factors, which integrate cell cycle progression with DNA repair and replication (Ren et al., 2002).

Taken together, our findings reveal that Gli3 controls the onset of cortical neurogenesis by regulating *Cdk6* expression levels and G1-phase length. Besides identifying *Cdk6* as Gli3's first target gene during corticogenesis, our results have major implications for cortical development in health as well as for human disease. Formation of the cerebral cortex requires a tight balance between proliferation and differentiation of RGCs to determine neuron numbers and, eventually, cortical size. A delayed onset of neurogenic divisions is predicted to lead to an increased number of radial units and to an overproduction of later-born cortical neurons at the expense of early-born neurons. These changes alter the size and laminar composition of the cortex, as observed in *Gli3*^cKO^ mutants, which is likely to affect cortical cytoarchitecture and function. Moreover, prolonged proliferation of cortical progenitor cells could not only underlie the macrocephaly in acrocallosal syndrome patients who carry mutations in *GLI3*, but also the agenesis of the corpus callosum in these patients (Bonatz et al., 1997; Elson et al., 2002; Philip et al., 1988; Schinzel, 1979). In *Gli3*^cKO^ mutants, the rostral midline becomes elongated and eventually starts to fold, with glial cells specifically forming at these folds but not in the rest of the cortex. The resulting ectopic glial fibres later interfere with midline crossing of callosal axons (Amaniti et al., 2013). Interestingly, such folds are still present in the cingulate cortex of *Gli3*^cKO^ postnatal animals (Amaniti et al., 2013). Similar glial abnormalities have also been observed in ciliary mouse mutants with callosal malformation (Benadiba et al., 2012; Laclef et al., 2015), suggesting that proliferation defects might also contribute to the malformation of the corpus callosum in the wider group of ciliopathies. Taken together, these wider considerations emphasize the importance of *Gli3* in governing the balance between proliferation and differentiation in cortical development.

## MATERIALS AND METHODS

### Mice

All experimental work was carried out in accordance with the UK Animals (Scientific Procedures) Act 1986 and UK Home Office guidelines. All protocols were reviewed and approved by the named veterinary surgeons of the College of Medicine and Veterinary Medicine, the University of Edinburgh, prior to the commencement of experimental work. *Flag-Gli3*, *Emx1Cre* and *Gli3^flox/flox^* mouse lines have been described previously (Blaess et al., 2008; Gorski et al., 2002; Nishi et al., 2015). *Emx1Cre;Gli3^flox/+^* and *Gli3^flox/flox^* mice were interbred to generate *Gli3*^cKO^ mutants. E0.5 was assumed to start at midday of the day of vaginal plug discovery and *Gli3^flox/flox^*, *Gli3^flox/+^;Emx1Cre* and *Gli3^flox/+^* embryos were used as controls. *Gli3^flox/flox^* animals were interbred with Tis21-GFP animals to generate *Gli3^flox/flox^;Tis21*^GFP/GFP^ mice. Double transgenic females were crossed with *Emx1Cre;Gli3^flox/+^* males to obtain *Emx1Cre;Gli3^flox/flox^;Tis21*^GFP/+^ conditional embryos. Transgenic animals and embryos were genotyped as described (Blaess et al., 2008; Gorski et al., 2002; Haubensak et al., 2004; Maynard et al., 2002; Ueta et al., 2002). For each marker and each stage, four to seven embryos were analysed.

For BrdU and IdU incorporation experiments, pregnant females were injected intraperitoneally with 100 mg of BrdU per gram body weight. Palbociclib was administered by oral gavage at 75 mg/kg body weight in lactate buffer (50 mM, pH 4.0) and animals were killed 24 h later.

### Immunohistochemistry and histology

Immunohistochemical analysis on 12 µm cryosections was performed as described previously (Theil, 2005) using antibodies against the following antigens (Table S2): rabbit anti-BrdU (1:50, Abcam, ab6326), mouse anti-BrdU (1:50, Becton Dickinson, 347580), rat anti-Ctip2 (1:1000, Abcam, 18465), chick anti-GFP (1:1000, Abcam, 13970), goat anti-Gli3 (1:200, R&D Systems, AF3690), rabbit anti-Pax6 (1:400, BioLegend, 901301), mouse anti-PCNA (1:500, Abcam, 29), rabbit anti-pHH3 (1:100, Millipore, 06-570), rabbit anti-phospho-Rb-S780 (1:200, Cell Signaling, 9307), mouse anti-Satb2 (1:200, Abcam, 51502), rabbit anti-Tbr1 (1:400, Abcam, 31940), rabbit anti-Tbr2 (1:1000, Abcam, 23345). Primary antibodies for immunohistochemistry were detected with Alexa-conjugated (1:200, Invitrogen) or Cy2/3-conjugated (1:100, Jackson ImmunoResearch) fluorescent secondary antibodies. The Gli3 and Tbr1 signals were amplified using biotinylated secondary IgG antibodies (Gli3: rabbit anti-goat IgG; Tbr1: swine anti-rabbit IgG) (1:400, Becton Dickinson) followed by Alexa Fluor 488 or 568 Streptavidin (1:200, Invitrogen). For counterstaining, TO-PRO-3 (1:2000, Invitrogen) or DAPI (1:2000, Life Technologies) were used.

For measuring S-phase lengths and total cell cycle lengths, pregnant females received a single, intraperitoneal injection of IdU, followed by an injection of BrdU 90 min later. Embryos were collected 2 h after the initial injection. To determine G2 length, pregnant females were treated with a single dose of BrdU, embryos were harvested 60, 90 or 120 min after BrdU administration and the proportion of BrdU^+^/pHH3^+^ cells was determined for each time point. Measurements of M-phase length involved determining the proportion of pHH3^+^/PCNA^+^ cells compared with the total number of PCNA^+^ cells. To determine the generation of neurons by pulse-chase experiments, pregnant females were intraperitoneally injected with BrdU. Embryos were harvested 24 h later and stained for BrdU and PCNA. The fraction of cells that had left the cell cycle and differentiated into neurons was calculated by dividing the number of BrdU^+^ PCNA^−^ cells by the total number of BrdU^+^ cells.

Quantifications of the dorsal surface area of the cortical hemispheres was obtained by outlining the cortex on images of whole brains. To calculate the length of the ventricular surface and cortical thickness lines were drawn on images of DAPI-stained coronal sections. Area and length measurements were performed using Fiji software.

### Western blot

Protein was extracted from the rostromedial telencephalon of E12.5 wild-type and *Gli3*^cKO^ embryos as described previously (Magnani et al., 2010). Protein lysates (30 μg) were subjected to gel electrophoresis on a 4-12% gradient NuPAGE Bis-Tris gel (Life Technologies), and protein was transferred to a PVDF membrane, which was incubated with rabbit polyclonal anti-Cdk6 antibody (1:300; Santa Cruz Biotechnology, sc-177) and mouse anti-GAPDH antibody (1:5000, Applied Biosystems, AM4300). After incubating with anti-rabbit IgG IRDye800CW (1:15,000, LI-COR Biosciences) and anti-mouse IgG Alexa Fluor 680 secondary antibodies (1:5000, Life Technologies), signal was detected using Odyssey Infrared Imaging System with Odyssey Software (LI-COR Biosciences). Values for protein signal intensity were obtained using Image Studio Lite Version3.1. Cdk6 protein levels were compared between wild-type and mutant tissue using a paired *t*-test.

### Electrophoretic mobility shift assays

For the GST-Gli3Zf construct, the cDNA encoding the Gli3 DNA-binding domain was PCR amplified and subcloned into the pGEX4-T3 vector using the *Sal*I and *Not*I restriction enzyme sites. Oligonucleotides used for cloning are summarized in Table S1. Electrophoretic mobility assays were performed with biotin-labelled oligonucleotides from the *Cdk6* promoter and purified GST and GST-Gli3-Zf proteins as described previously (Hasenpusch-Theil et al., 2017). The binding reactions were separated on native 5% acrylamide gels and transferred onto positively charged nylon membranes (Roche) with a Perfect Blue Semi-dry electro blotter (60 min at 120 V, 5 mA). After UV crosslinking, biotin-labelled probes were detected using a Chemiluminescent Nucleic Acid Detection Module (Thermo Fisher Scientific) according to manufacturer's instructions and imaged using a Kodak BioMaxXAR film. For oligonucleotide sequences covering the wild-type or mutated Gli3-binding sites, see Table S1. The exchanged nucleotides in the mutated forms are underlined. Wild-type and Gli3-binding site mutant oligonucleotides were used as specific and non-specific competitors, respectively, in a 10- to 100-fold molar excess.

### Luciferase assay

Genomic DNA fragments from the *Cdk6* promoter were generated via PCR with the oligonucleotides listed in Table S1 from BAC clone RP23-53P17, subcloned using pCR-Blunt II-TOPO cloning kit (Invitrogen) and verified by sequencing. A 3.35 kb *Cdk6* promoter fragment was subcloned into the pGL4.10 promoterless firefly luciferase reporter vector (Promega) after *Asp*718 and *Xho*I restriction digests. HEK293 cells were transfected using Lipofectamine 2000 (Invitrogen). Gli3 repressor and GFP were expressed from previously described pCAGGS expression vectors (Persson et al., 2002). The *Renilla* luciferase vector was p*RLSV40* (Promega). HEK293 cells were harvested 48 h after transfections and analysed with the Dual Luciferase Reporter Assay System (Promega).

### RNAseq, qRT-PCR, ChIPseq and bioinformatic analyses

For RNAseq experiments, rostromedial dorsal telencephalic tissue was dissected from E11.5 and E12.5 embryos and pooled (E11.5: six tissues; E12.5: three tissues) to generate four different replicates per genotype (control: *Emx1Cre;Gli3*^flox*/+*^; mutant: *Emx1Cre;Gli3*^flox/flox^). Total RNA was extracted using RNeasy Plus Mini Kit (Qiagen). After assessing the integrity of the RNA samples with an Agilent 2100 Bioanalyzer, (RIN>8), all RNAs were further processed for RNA library preparation and sequenced (paired-end, 50 bp/100 bp reads) on an Illumina SOLEXA GAII platform at Edinburgh Genomics (University of Edinburgh). Sequence reads were aligned with NCBI build 38 for *Mus musculus* and analysed using Genomatix Software Suite (Genomatix, Munich) based on the ElDorado 12-2013 database and Genomatix Genome Analyzer (GGA). The number of differentially expressed genes was determined using Ensemble as the source of transcripts and DeSeq 1.10.1 as the differential expression method with a threshold *P*-value ≤0.05.

Differentially expressed genes were analysed for gene ontology using Genomatix Pathway Systems (GePS) and GeneRanker (Berriz et al., 2003). GePS allows networks to be created based on literature, and GeneRanker includes more in-depth filtering with Gene Ontology or Genomatix proprietary annotation. To identify putative Gli3-binding sites in the 3.35 kb upstream promoter region of *Cdk*6, the sequence was run through MatInspector of the Genomatix Software using the Gli3 weight matrices V$GLI3.01 AND V$GLI3.02. Identified binding sites were ranked by matrix similarity scores and the highest ranked sequences were chosen for further analysis. To validate differential expression of *Cdk6*, total RNA was isolated from the rostromedial telencephalon of E11.5 and E12.5 control and *Gli3*^cKO^ embryos using an RNeasy Plus Micro Kit (Qiagen) and reverse transcribed using ImProm-II Reverse Transcriptase (Promega). Quantitative reverse transcription PCR (qRT-PCR) was performed using QuantiTect SYBR Green (Qiagen) and a DNA Engine Opticon System (GRI); the oligonucleotides used are summarized in Table S1. For each sample, Ct values were extrapolated using the Opticon software and ratios of relative gene expression levels of β-actin (reference gene) and *Cdk6* were calculated based on a modified ΔΔCt method taking into account different PCR kinetics (Pfaffl, 2001); PCR efficiencies are summarized in Table S3.

Chromatin immunoprecipitation followed by sequencing (ChIP-seq) was performed on E12.5 cerebral cortices derived from Flag-Gli3 mice (Nishi et al., 2015) using a Millipore Magna ChIP A/G kit and anti-Flag M2 antibody (Sigma) on 1×10^6^ cells. Two independent ChIP experiments were sequenced on an Illumina Hiseq 2000 platform. Illumina sequencing adapters were trimmed off with Trimmomatic v0.36 software and ChIP-seq reads were aligned to the genome using Bowtie2. The MACS2 (model-based analysis of ChIP-seq) programme was used for peak calling with mfold values 150-200 and q-value cutoff at 0.001. For graphical illustration, Gli3 ChIP samples were normalized to input samples using bamCompare.

### Statistical analyses

Data were analysed using GraphPadPrism 6 software with *n*=3-7 embryos for all analyses. Power analysis of pilot experiments informed minimum samples size. Mann–Whitney tests were in general performed for immunohistochemical analyses and for measurements of cortical surface area. The distribution of Tbr1^+^, Ctip2^+^ and Satb2^+^ neurons was analysed using a two-way ANOVA followed by Sidak's multiple comparisons test. For the palbociclib rescue experiment, ANOVA with Tukey's multiple comparisons test was performed (*P<0.05, **P<0.01,***P<0.001). For quantifications of the palbociclib rescue experiment, the observer was blinded to the experimental treatments. Owing to morphological changes, blinding was not possible for experiments involving *Gli3*^cKO^ embryos older than E11.5. In these cases, scores were validated by a second independent observer.

## Supplementary Material

Supplementary information
