# Supplementary material for: Gli3 controls the onset of cortical neurogenesis by regulating the radial glial cell cycle through Cdk6 expression
Source: Development. 2018 Aug 20;145(17):dev163147. doi: 10.1242/dev.163147 (PMC6141774; doi:10.1242/dev.163147)
Supplement: Supplementary information [file develop-145-163147-s1.pdf]

Supplementary Figure 1: Gli3 expression in cortical progenitors and in *Gli3*^{cKO} embryos. (A, E) Gli3 protein is co-expressed with Pax6 in radial glial cells in the rostromedial (A) and lateral telencephalon (E). (B-D, F-H) Gli3 is transiently expressed in Tbr2+ basal progenitor cells. In the lateral telencephalon, basal progenitors in the deep ventricular zone express Gli3, while Tbr2+ cells in upper positions express little or no Gli3. Arrows demarcate Gli3+Tbr2+ basal progenitors, arrowheads Gli3-Tbr2+ cells. (I-L) Gli3 protein is expressed in the dorsal telencephalon and in the lateral ganglionic eminence (LGE) of E11.5 (I, J) and E12.5 (K, L) embryos and shows a dorsal to ventral expression gradient in the thalamus (th) (L, P). (M, N) In E11.5 *Gli3*^{cKO} embryos, Gli3 protein is still present in the rostrolateral cortex while it is absent at more caudal levels. (O, P) The E12.5 cortex lacks Gli3 protein while expression in the CGE, LGE and thalamus is not affected. Scale bars: 50μm (A, E); 200μm (I).

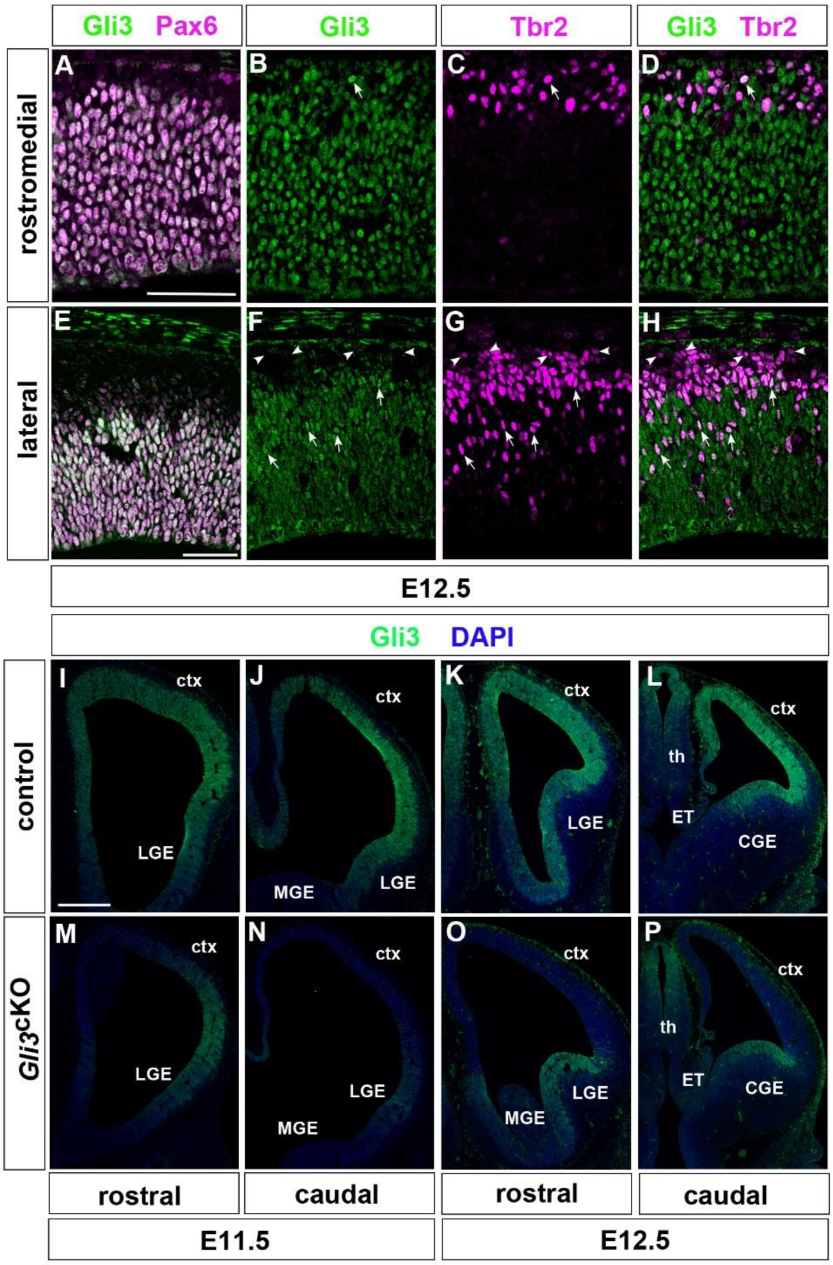

Development • Supplementary information

Supplementary Figure 2: The rostral telencephalon is enlarged in E12.5 *Gli3*^{cKO} embryos. (A, B) Whole mount view of the embryonic heads of a control (A) and a *Gli3*^{cKO} embryo (B). Arrows indicate the prominent bulging of the mutant forebrain. Scale bar: 1mm.

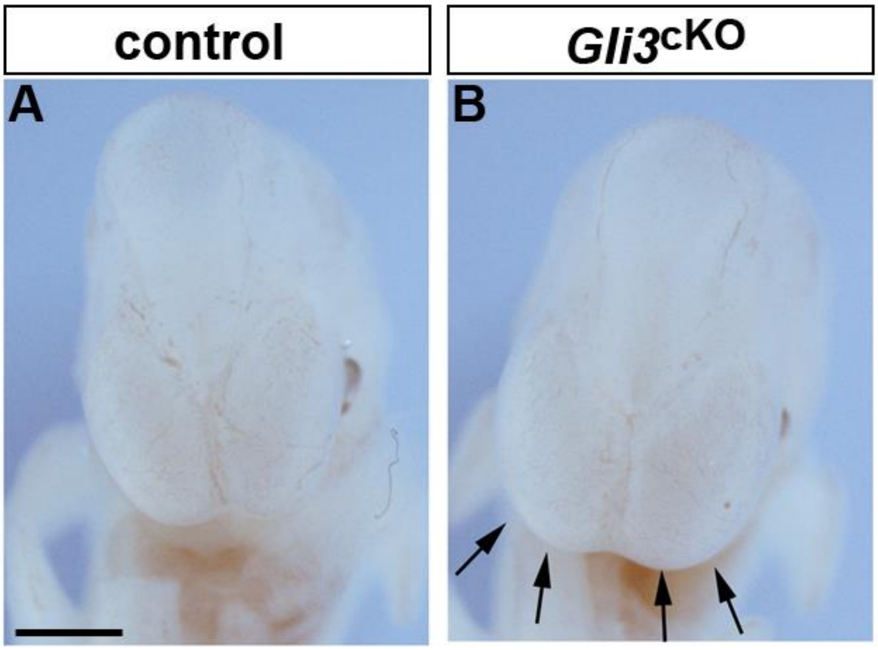

Development • Supplementary information

Supplementary Figure 3: Morphological abnormalities in the rostromedial telencephalon of *Gli3*^{cKO} mutants. (A, B) Coronal brain sections through the rostral telencephalon of E12.5 control (A) and *Gli3*^{cKO} (B) embryos stained with DAPI. The yellow line in (A) indicates the measurement of midline length. (C) Quantification of midline length. (D, E) Measurements of cell numbers in a 175 μm wide box in E11.5 (D) and E12.5 (E) embryos. All statistical data is presented as means ± 95% CI; Mann Whitney tests; n = 6 (C); n=7 (D) and n=5 (E); * $p < 0.05$; ** $p < 0.01$. ctx: cortex; LGE: lateral ganglionic eminence; sep: septum. Scale bar: 250 μm.

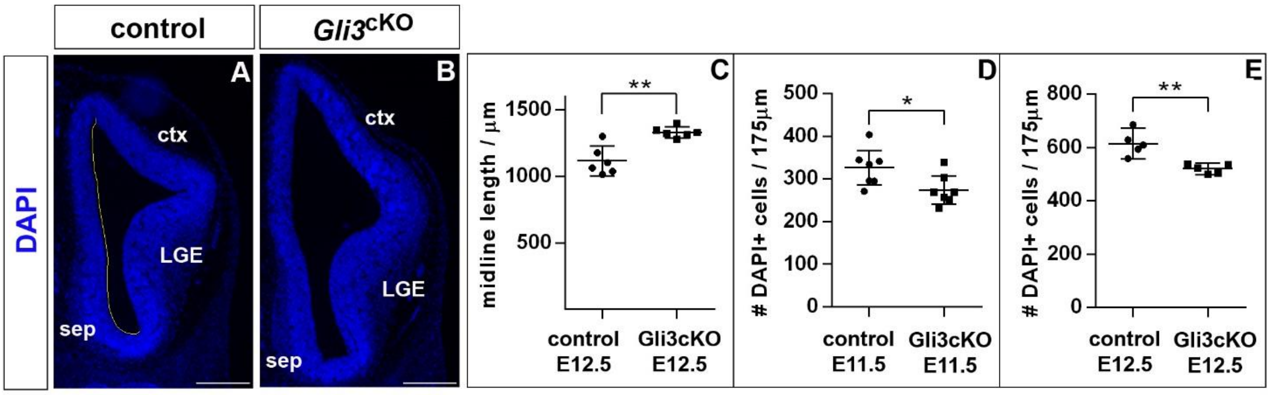

Supplementary Figure 4: Enlarged ventricular surface in *Gli3*cKO mutants. (A, B) Coronal sections through the brains of an E17.5 control and *Gli3*cKO embryo. Yellow lines indicate measurements of the dorsal telencephalic ventricular surface and cortical thickness. (C, D) Statistical evaluation of surface length (C) and thickness (D) measurements. All statistical data is presented as means ± 95% CI; Mann Whitney tests; n = 5; * $p < 0.05$; ** $p < 0.01$. CC: corpus callosum; ctx: cortex; Pb: Probst bundle; sep: septum. Scale bar: 500 μm.

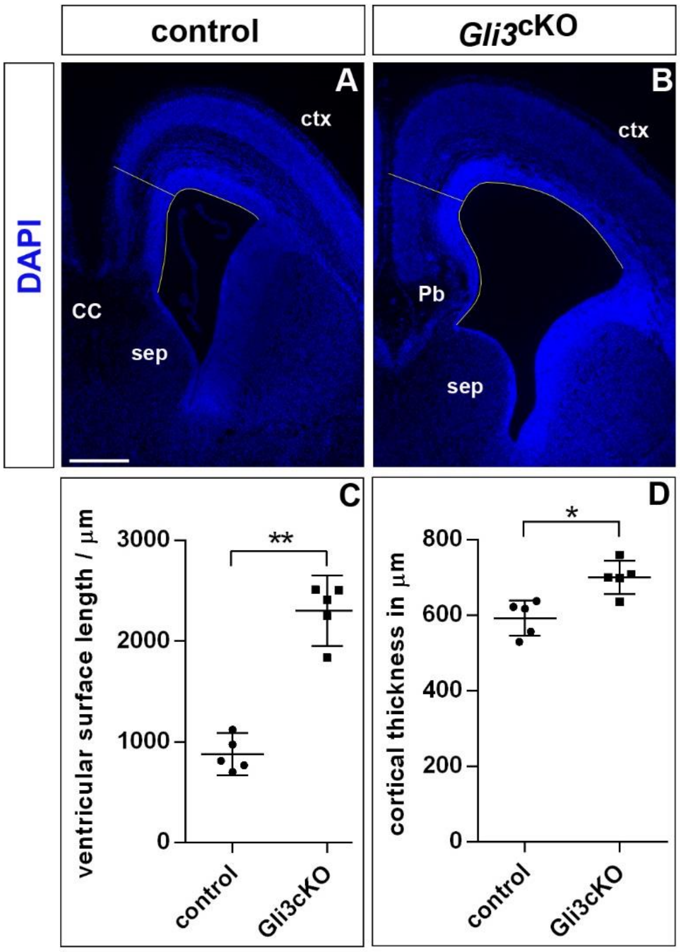

Development • Supplementary information

Supplementary Figure 5: Numbers of cortical neurons in *Gli3*^{cKO} mutants. Quantifications of Tbr1+ (A), Ctip2+ (B) and Satb2+ (C) neurons. All statistical data is presented as means ± 95% CI; Mann Whitney tests; n = 4; * $p < 0.05$.

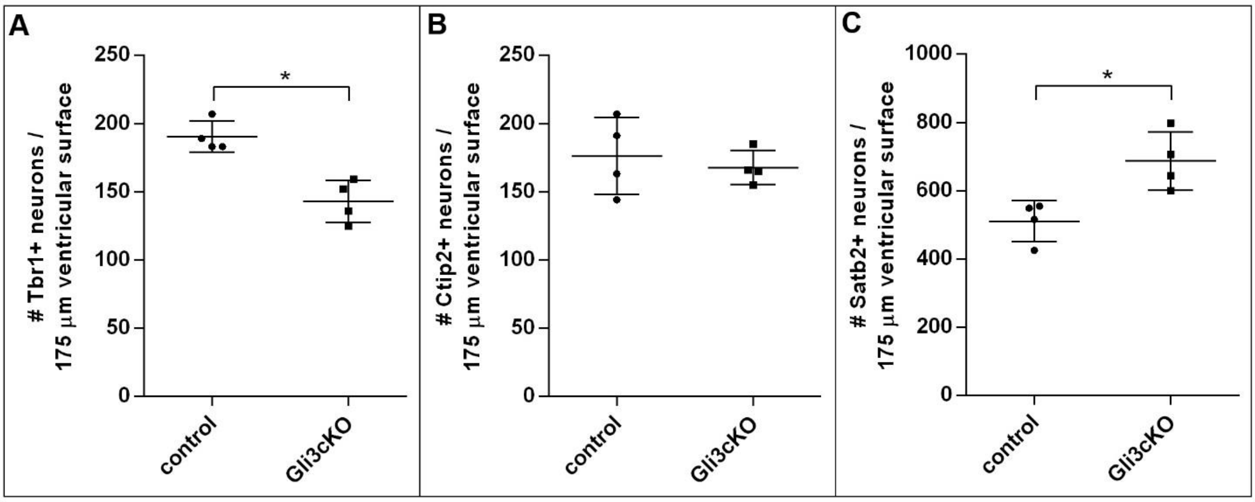

Development • Supplementary information

Supplementary Figure 6: Gene ontology (GO) analysis of genes differentially regulated in E11.5 (*A*) and E12.5 (*B*) cortical progenitor cells of *Emx1Cre*;*Gli3*$^{fl/fl}$ mutants. The *X*-axis values correspond to DAVID *p*-values. All terms reported have a false discovery rate (FDR) < 5%.

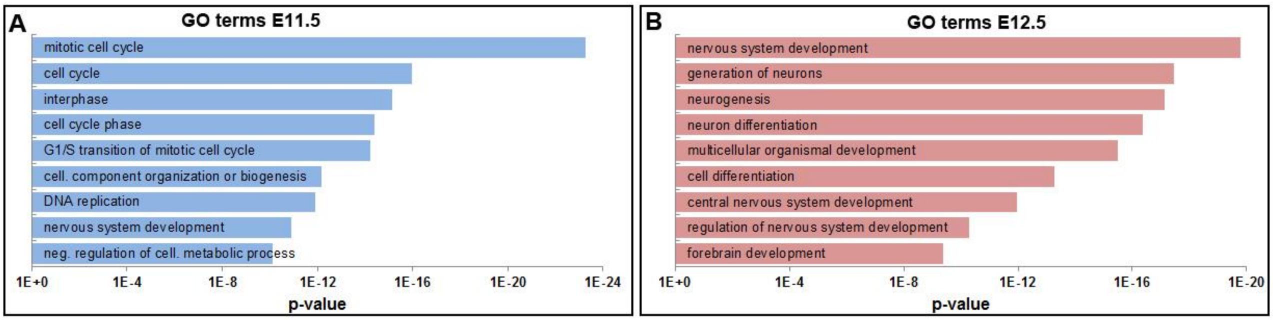

Development • Supplementary information

Supplementary Figure 7: Proportion of proliferating vs differentiating in *Gli3*^{cKO} mutants. (A, B, E, F) Double immunostaining for Pax6 and GFP to distinguish proliferating (Pax6+GFP-) from differentiating (Pax6+GFP+) radial glial cells in *Gli3*^{cKO} mutants positive for the *Tis21-GFP* transgene. (I) Quantification of the Pax6/GFP immunostainings. The proportion of proliferating radial glial cells is decreased in E12.5 *Gli3*^{cKO} embryos (C, D, G, H) Coronal sections immunostained for Tbr2 and GFP showing proliferating (Tbr2+GFP-) and differentiating (Tbr2+GFP+) basal progenitors in *Gli3*^{cKO} mutant, *Tis21-GFP*+ embryos. (J) Quantification revealed an increased proportion of differentiating basal progenitors in E11.5 *Gli3*^{cKO} mutant embryos. All statistical data is presented as means ± 95% CI; Mann Whitney tests; n = 4 except for (C, D) where n=5; * $p < 0.05$. Scale bars: 50 µm.

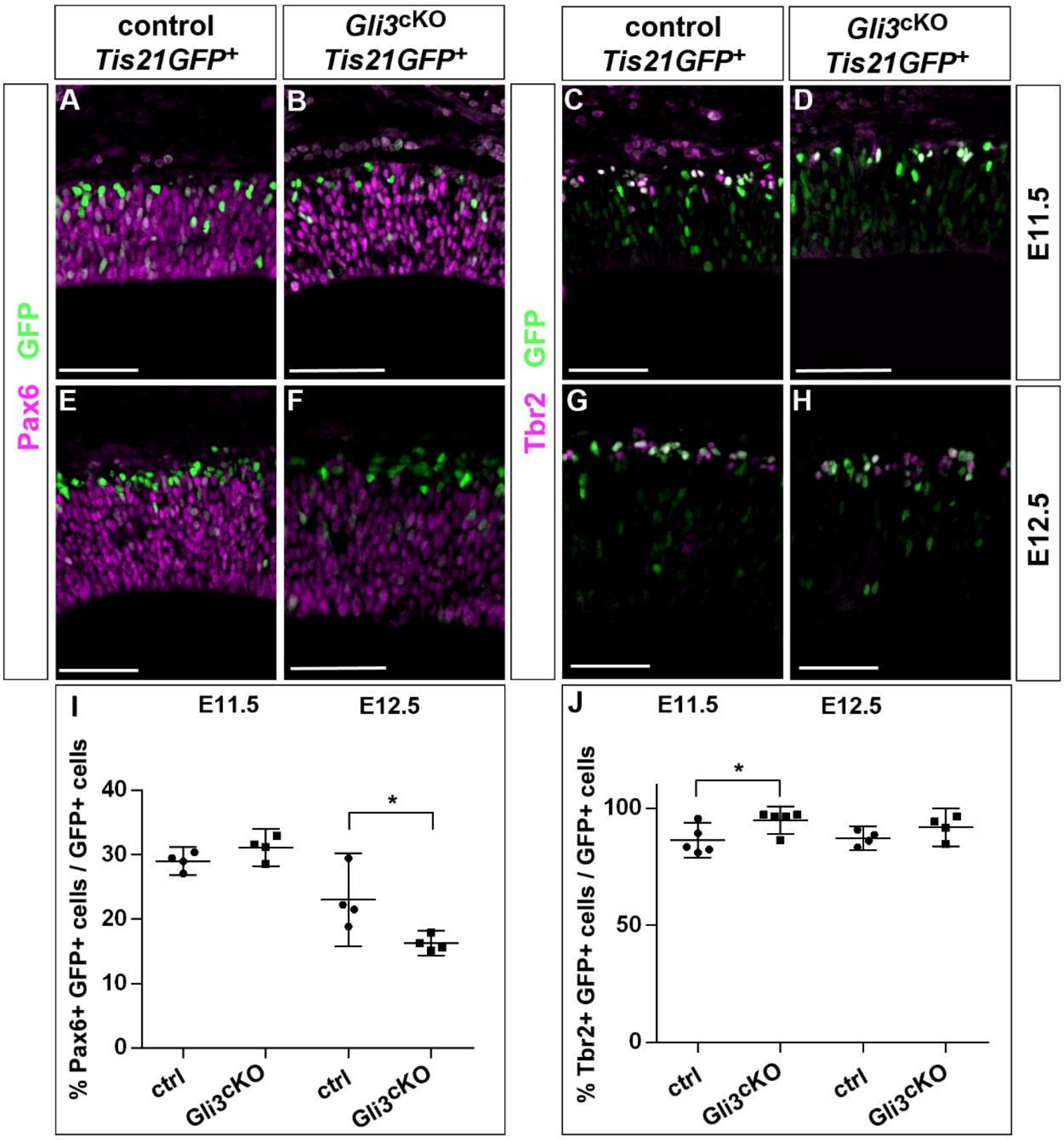

Development • Supplementary information

Supplementary Figure 8: Neuron formation in the cortex of E12.5 *Gli3*^{cKO} mutants after Palbociclib administration at E11.5. (A-D) Immunohistochemistry for Tbr1 and BrdU revealing the proportions of newly formed neurons in E12.5 control and *Gli3*^{cKO} embryos after BrdU and lactate or Palbociclib administration 24h earlier. Arrows indicate Tbr1+BrdU+ neurons (E) Quantifications of the immunostainings in (A-D). Statistical data is presented as means ± 95% CI; n = 5; * $p < 0.05$; Kruskal-Wallis test with Dunn's multiple comparisons test. Scale bar: 50 μm.

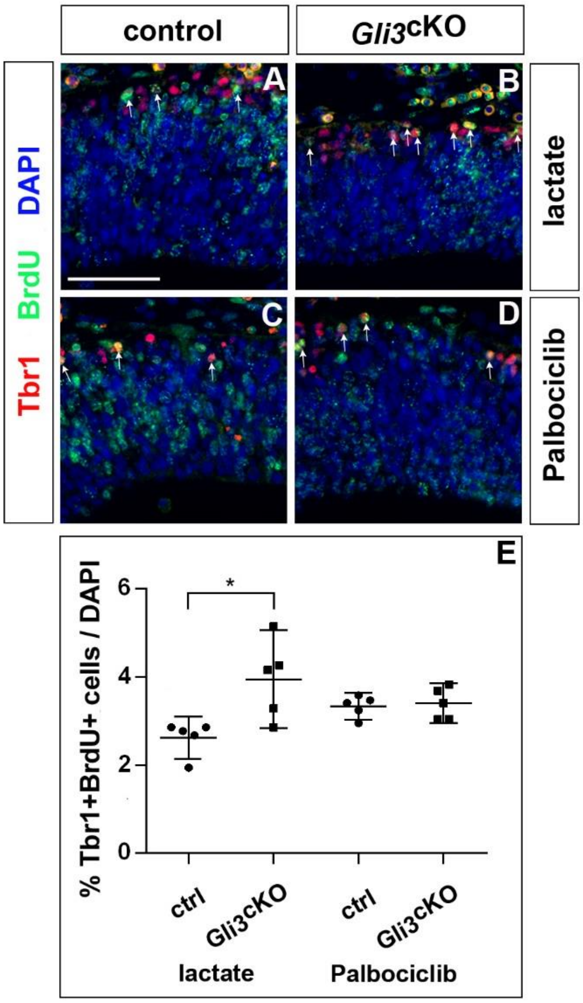

Development • Supplementary information

Supplementary Table 1: Oligonucleotides used in this study

**oligonucleotide**	**5' - 3' sequence**	**restriction site used for subcloning**
**EMSA**		
Cdk_GliBS1_F	GACTCACGTGGGTGGGCTGAGGG	
Cdk_GliBS1_R	CCCCTCAGCCCACCCACGTGAGTC	
Cdk_GliBS1m_F	GACTCACGTGGGTATGCTGAGGG	
Cdk_GliBS1m_R	CCCCTCAGCATACCCACGTGAGTC	
Cdk_GliBS2_F	GGGGGGCTGTGTGGCCCCTCGGAATC	
Cdk_GliBS2_R	GATTCCGAGGGGCCACACAGCCCCC	
Cdk_GliBS2_F	GGGGGGCTGTGTATCCCCTCGGAATC	
Cdk_GliBS2m_R	GATTCCGAGGGGATACACAGCCCCC	
**GST-Gli3_Zn finger cloning**		
Gli3ZF_Fw	AAAGTCGACCAGGAGCCTGAAGTCATCTACG	SalI
Gli3ZF_Rev	AAAGCGGCCGCGTCCCCACGCTGCTTCTTGG	NotI
**Cdk6 promoter cloning**		
Cdk6_F1	AAAGGTACCCCGCCTCACCCTGTCAACAC	Asp718
Cdk6_R1	AAACTCGAGGCTGGCTTCAGGCTGCGGG	XhoI
**qRT-PCR**		
CDK6_mRNA	ATGTTTCGCAGAAAGCCTCTT	
CDK6_mRNA	GTCCCTAGGCCAGTCTTCCT	
Actb_F	ACTATTGGCAACGAGCGGTT	
Actb_R	AGCACTGTGTTGGCATAGAGGT	

Development • Supplementary information

Supplementary Table 2: Antibodies used in this study

Antibody	Antigen	Dilution	Raised in	Source (Catalog #)	Validation references
α-BrdU	bromodeoxyuridine [BU1/75(CR1)]	01:50	rabbit	abcam (ab6326)	
α-BrdU	bromodeoxyuridine (clone B44)	01:50	mouse	Becton Dickinson (347580)	
α-Ctip2	*H. sapiens* (aa1-150) [clone 25B6]	1:1000	rat	abcam (ab18465)	
α-Flag	synthetic peptide DYKDDDDK	0.27mg/ ml	mouse	Sigma (F3165)	
α-GFP	E. coli-derived recombinant full length protein corresponding to GFP	1:1000	chicken	abcam (ab13970)	
α-Gli3	E. coli-derived recombinant human GLI3 (aa1-479)	1:200	goat	R&D Systems (AF3690)	This work (Fig. S1)
α-Pax6	peptide:(QVPGSEPDMSQYWPRLQ) derived from C-terminus of mouse Pax-6	1:400	rabbit	Biolegend (901301)	
α-PCNA	Protein A-rat PCNA fusion obtained from pC2T [PC10]	1:500	mouse	abcam (ab29)	
α-pHH3	linear peptide corresponding to human Histone H3 at Ser10	1:100	rabbit	Millipore (06-570)	
α-Phospho-Rb (Ser780)	synthetic phosphopeptide corresponding to residues surrounding Ser780 of human Rb	1:200	rabbit	Cell Signaling (9307)	
α-Satb2	recombinant fragment corresponding to Human SATB2 (C terminal)	1:200	mouse	Abcam (ab51502)	
α-Tbr1	synthetic peptide conjugated to KLH derived from within aa50-150 of mouse TBR1	1:400	rabbit	abcam (ab31940)	
α-Tbr2	synthetic peptide conjugated to KLH derived from within residues 650 to the C-terminus of mouse TBR2/Eomes	1:1000	rabbit	abcam (ab23345)	

Development • Supplementary information

Supplementary Table 3: Differentially expressed genes in E11.5 and E12.5 *Gli3*^{cKO} mutants.

Click here to Download Table S3

Supplementary Table 4: Summary of statistical analyses

Click here to Download Table S4
